# Participatory Action Research Approach to improve Adolescent Girls' Reproductive Health

**DOI:** 10.5935/1518-0557.20200034

**Published:** 2020

**Authors:** Zahra Bostani Khalesi, Abdolhosein Emami Sigaroudi, Rabiollah Farmanbar

**Affiliations:** 1 Social Determinants of Health Research Center, Guilan University of Medical Sciences, Rasht, Iran; 2 Cardiovascular Diseases Research Center, Department of Cardiology, Heshmat Hospital, School of Medicine, Guilan University of Medical Sciences, Rasht, Iran

**Keywords:** adolescent, participatory action research, reproductive health

## Abstract

**Objective:**

School-based reproductive health education programs (RHEP) motivate adolescent girls to maintain and improve their health, and prevent diseases. The purpose of this PAR was to design, implement and evaluate RHEP to strengthen adolescent girl's reproductive health.

**Methods:**

The PAR process was selected as an RHEP strategy, and it has four main phases, including: 1) assessment to explore the reproductive health education needs (RHEN) of adolescent girls. We collected data through in-depth individual interviews with 11 adolescent girls (12-18 years) in high school, 2 focus group discussions (FGD), and 4 interviews with the key informants. 2) Intervention design involved a Delphi approach to design an intervention that would address each need using 7 expert participants with a background in primary health, health promotion and other youth-focused professions. 3) In the action plan phase, the workshops, lecture meetings, counseling, and FGD were organized by the research team. 4) The impact of the intervention was evaluated through a mixed evaluation methodology, a semi-structured interviews with stakeholders and key informant, quasi-experimental assessment and FGD.

**Results:**

There were three themes we extracted from the data: (a) the need for RHP for adolescent girls, (b) sources of information about RH, and (c) the need to empower teachers to provide RHEP to their students. a) Workshops, (b Counseling, c) Lecture Meetings, d) Focus Group. The study showed that the level of knowledge about RH in more than half of the participants was poor and only in nearly half of them it was moderate.

**Conclusion:**

Results suggest that Iranian adolescents do not have adequate education regarding RH, and RHEP by PAR can be effective in improving the knowledge and behavior of female students.

## INTRODUCTION

The period of adolescence is a life phase in which young people are particularly vulnerable to health risks. WHO defines 'Adolescents' as young people between the ages of 10 and 19 years (WHO, 2017). During teenage years, sexual development and the ability to reproduce occur, but most adolescents go through this time with only ambiguous information on RH (Eslamimehr *et al*., 2016).

Given that adolescent girls are future mothers, information about RH is important not only to improve their health but also to prevent possible health problems (Mirzaii & Olfati, 2014). Adolescents become vulnerable to problems due to a lack of knowledge and skills to avoid high-risk behaviors and lack of acceptable and appropriate RH information and services (Silva, 2015). RH refers to the health and well-being of women and men in terms of sexuality, pregnancy, birth, and their associated conditions, diseases, and illnesses (Denno *et al*., 2015). In 1994, the International Conference on Population and Development (ICPD) focused on the importance of adolescence to RH throughout one's life span (Obong'o & Zani, 2014).

At present, the world is more interlinked than ever before, high school students all over the globe are demanding accurate and accessible information about RH (Shakour *et al*., 2016). They need not only information about bodily processes and a better understanding of society's set of behaviors, but they also need the skills necessary to develop healthy relationships and engage in responsible decision-making, especially during adolescence when their emotional development accelerates (Silva, 2015; Obong'o & Zani, 2014). These adolescents need scientific-based information to raise their RH awareness and prevent RH-related problems (Inter-agency Working Group on Reproductive Health in Crises, 2010). Provision of such knowledge should be internationally regarded as one of the human rights (Silva, 2015). School-based RHEP have the potential to be an effective and cost-effective way to reduce puberty problems associated with education as it informs adolescents on safe behavior, abstinence and to be more responsible in making decisions about their lives (Casey, 2015). Schools, as a learning environment, play a vital role in the development of young people by providing knowledge and skills, and have been recognized as an appropriate setting for health promotion, including RH (Eslamimehr *et al*., 2016).

In addition to be a setting where adolescents spend most of their time, schools have the infrastructure in place for health activities, serving as a link between young people and their local communities (Silva, 2015). Therefore, governments and other stakeholders have the responsibility of developing programs that provide adolescents with accurate information to enable them to have desirable control over issues pertaining to RH (Casey, 2015). In addition, these should seek to provide RH services that are consistent with the community's cultural and religious values (Obong'o & Zani, 2014).

In Iran, the concept of RH has not been well understood and it is highly controversial (Jalali Aria *et al*., 2010). Because of the shame, taboos, cultural and social beliefs, an appropriate RHEP for adolescents has been neglected in the family setting and school (Simbar *et al*., 2017). Besides, these issues are not discussed into textbooks (Jalali Aria *et al*., 2010). Despite reported increases in global connections and media coverage, Iranian youth are still facing RH problems (Shakour *et al.,* 2016). Many adolescents lack awareness of available services; they are also restricted from seeking RH information and services and fear the stigma associated with seeking RH care from formal healthcare centers (Madanipour *et al*., 2009). These facts show that there is a strong need to assess adolescent knowledge and there are plans for strategies to help them address knowledge gaps, since risk-related behaviors among adolescents can be reduced through raising awareness about HIV/AIDS, and related issues (Obong'o & Zani, 2014). Since meaningful girl engagement can ensure that girls' voices are heard, their ideas are welcomed, and their knowledge is used in developing and implementing sustainable and effective programs, research, and policies (Nair *et al*., 2012).

Participatory Action Research (PAR) is a means for school psychologists, as participatory researchers, to provide collaborative research leadership within schools for the development of system-wide interventions (Hawkins, 2015). A case study indicated that the PAR process resulted in the design and implementation of a school-based research partnership program that was culturally specific, acceptable to stakeholders. The purpose of this participatory action research study was to design, implement and evaluate RHEP.

## MATERIALS AND METHODS

PAR was selected as an RHEP strategy, with a twofold goal: to develop knowledge and effective interventions. The PAR approach, as described by Maguire (1987), provided the methodological framework for this study (Dickson & Green, 2001).

The project lasted for approximately 2 years after the protocol was accepted and became financially supported in September 2018. The approach had four main phases, included in: 1) Needs assessment, 2( Intervention design, 3) Action planning, 4) Evaluation of the intervention.

### Phase 1: Needs Assessment

In this phase, we explored the reproductive health educational needs (RHEN) of adolescent girls. Two main data collection strategies were employed in this phase; FGD and in-depth interview. Phase one of this project was conducted using conventional content analysis. Participants were selected through purposive sampling. We collected the data through in-depth individual interviews with 11 adolescent girls (12-18 years) in high school, 2 FGD, and 4 interviews with the key informants. The interview guide contained elementary questions and was developed by researchers, and the expert researchers in the field of RH confirmed its validity.

All interviews were recorded and transcribed. We analyzed the transcribed texts in five steps. In the first step, the interviews are read through and listened to several times. In the second step, we assessed meaning units related to the aim. In the third step, the meaning units are condensed, labeled, and finally coded based on their content. Based on the codes, subcategories and categories are developed in the fourth step. In the fifth step, the categories were carefully discussed and the main categories identified. We analyzed the data using content analysis method and the MAXQDA software. To complete the RHEN concept and dimensions, we performed an extensive review of the related literature in Science Direct, PubMed (include in Medline), and Google Scholar, and some Persian databases including Irandoc, and Medlib. We included studies meeting the following criteria: Studies that explored RHEN, Published in English and Persian language. Published qualitative, mixed methods, or quantitative studies. The study evaluated a school-based RHE intervention delivered in Iran. We excluded those studies in which the topic was irrelevant to RH.

### Phase 2: Interventions Design

The purpose of this phase was designing need- based interventions to improve adolescent girls' reproductive health. Need-based intervention is an effective strategy for improving health literacy (Ramezanzadeh *et al*., 2010). We used a Delphi approach to design an intervention that would address each need using participants' suggestions. There is a lack of consensus on what represents adequate sample size for Delphi studies. The Delphi technique is a group communication process as well as a method for achieving opinion consensus. The Delphi panel size does not depend on statistical power, but relies on the dynamics of a group for arriving at a consensus with the literature recommending 10-18 experts on a Delphi panel (Shariff, 2015). Data collected in the first round of Delphi questionnaires will be qualitative in nature and will be analyzed using content analyses techniques (Donohoe *et al*., 2012).

During this phase, the study engaged 7 expert participants with a background in primary health, youth work, health promotion and other youth-focused activities. We employed several measures to increase the rigor of the study. To reduce bias, we collected the data, coded it and discussed regularly with the research team. We managed to Increase credibility through prolonged engagement with the setting and regular checking raw data, analyses and reports. Detailed descriptions of the contextual data and activities of the study, through immersion, reflective journaling and detailed documentation presented enabled others to analyze the situation and research outcomes based on context. To reduce bias and enhance conformability, the research team analyzed the coding and themes.

### Phase 3: Action Planning

This phase involved action planning and project implementation. The goals, policies and proposed actions detailed in the previous phase (Delphi) lead to project implementation activities. In this phase, we developed the documents to implement specific goals and objectives (actions) identified in the RHEP through task development. The tasks in the action plan, establish in details how who shall conduct the activities, when, and within what time frame and funding authority. The stakeholders organized the workshops. The research team (adolescence committee members) began the lecture based on the overall aim of the training session and on the specific learning objectives. They presented a description of what would be covered and the sequence to be followed. Materials included handouts and briefing papers. We used flipcharts, overhead projectors, blackboards, and video equipment. FGD was used to assess the values expressed by participants, the range of knowledge and to identify issues in which there is agreement or disagreement between members.

### Phase 4: Intervention Assessment

This phase's aim was to evaluate the RHEP using a mixed evaluation methodology include semi-structured interviews with stakeholders and key-informants, quasi-experimental assessment, FGD.

The fourth phase, the implementation stage of carrying out the interventions and the evaluation, represents one important empirical part of the RHEP. In this phase, useful data would be pre-post evaluation tests, both summary scores for evidence and context, narrative summary, and evaluation of facilitation approach, according to the PAR.

Before and after the intervention, participants in both the intervention and control groups completed a questionnaire concerning their knowledge about RH. The researchers developed the self-administered questionnaire based on an extensive literature review, as a tool for data collection. The questionnaire consisted of two sections. The first section related to socio-demographic items such as age, gender, educational level, occupational status of parents, and monthly family income. The second section was designed to assess participants' knowledge on RH through 18 knowledge-testing items (i.e., physical and psychological changes during puberty, menstruation, misconception regarding menstruation, hygiene practices during menstruation, mode of HIV transmission and prevention). All the questions were explained to the participants and they were asked to fill in the answers carefully. Knowledge-test questions were multiple choice. A score of one was given to the correct answers and zero to the wrong answers. The knowledge scores ranged from 0 to 18 with a higher score representing better knowledge about RH. The level of knowledge was described as good if they scored 12-18, moderate if 6-11, and poor if 0-5.

Fifteen students and 10 university experts in RH and health education determined the quantitative face validity of the questionnaire. The face validity of the item was calculated by using the Impact Score equation. The Content Validity Index (CVI) and the Content Validity Ratio (CVR) were computed for the whole test. In the current study, the calculated CVI and CVR were 0.89 and 0.91, respectively. We measured the reliability using Cronbach's alpha method, at a value of 0.81. This result demonstrated that the questionnaire was internally consistent.

An independent sample t-test was used for statistical analysis. The results were expressed as frequency, mean, and standard deviation (SD) for qualitative and quantitative data, respectively. We analyzed the data using the SPSS, version 21. A value of *p*<0.05 was considered statically significant.

In order to protect the rights of the participants, the research team explained the purpose of the study, assured participants that their information would be private and confidential, and explained that participants could withdraw from the study at any time. All adolescent girls who participated in the study signed written informed consent forms.

## RESULTS

Our action plan enabled us to track, measure, monitor and adjust our stakeholder engagement efforts. Our findings in the first phase (Reproductive health education needs of adolescent girls) pointed to three main themes: the necessity of RHEP for adolescent girls, sources of information about RH and the need to empower teachers to provide RHP to their students. Most participants believed in limiting RHEP for adolescent girls; they stated that teachers are the best people to educate adolescents about RH. Each of these included various sub-themes.

The different stakeholders arrived at a set of solutions through an iterative process of consultation and reaching the agreement. Based on the findings of the second phase (interventions, design), appropriate and culturally based content for participants included reproductive anatomy, reproductive biology, menstrual hygiene, HIV, and reproduction organ cancer prevention. In addition, we identified themes relevant to the teaching method in this phase.

All expert panel members in the Delphi panel agreed with multilevel interventions that included: a) Workshops, b) Counseling, c) Lecture Meetings, d) FGD.

In the third phase of this study (Action Plans), the findings clearly demonstrated that for the successful implementation of RHEP we needed quality teaching and participatory teaching methods that engaged students. The stakeholders planned, organized, promoted, and conducted their separate workshops. These participants chose the topics and selected speakers among themselves. Verbal feedback from the attendees was positive. In addition, the written feedback indicated that FGD helped develop a better understanding of RHEP.

According to our study's findings, the participants' awareness of RH issues was inadequate. In the final evaluation, all methods that presented the action phase included the workshops, lecture meetings, counseling, and FGD. They were satisfactory, but the most effective method was FGD, from the students' point of view.

## DISCUSSION

This study used the potential of PAR as an approach to create knowledge and empower RH among adolescent girls.

PAR provides an opportunity to bring adolescents together and share their experiences with the aim of promoting reproductive health. Sharing experiences gave the adolescents a sense of collective identity. Sharing these feelings assured them that they were not alone (Frahsa *et al*., 2014).

PAR managed to enable the adolescents' abilities through cooperation with others, challenging the status quo, being creative in interaction spaces, and practicing opportunities (Dickson & Green, 2001). On the other hand, the ‘competition' in practice to better planning, coordinate and implement activities cause empowers students (BeLue *et al*., 2012).

Overall findings revealed that participants had many unmet RHEN and PAR needs, which are effective in improving adolescents' knowledge regarding RH issues. Adolescents have the right to obtain accurate and sufficient information about RH to make responsible and correct decisions regarding their RH needs (Mirzaii Najmabadi *et al*., 2018). There are no explicit policies supporting the provision of RH education for them in Iranian schools. For the successful implementation of the school-based component of this intervention, participatory teaching methods that engage students are necessary (Panjalipour *et al*., 2018). Also, the healthcare system in Iran does not provide RH services tailored for adolescents. In fact, many reproductive healthcare services are provided only for married people.

The current study revealed that participants do not have enough, or correct knowledge about RH. This problem was reported in several Iranian studies (Simbar *et al*., 2017; Mirzaii Najmabadi *et al*., 2018; Panjalipour *et al*., 2018).

Yazdi *et al*. (2006) revealed that 43% of adolescents agreed that HIV/AIDS is curable if diagnosed at an early stage, and 81% of participants said that HIV/AIDS is a vaccine-preventable-disease. General awareness concerning the physical changes that occur during adolescence is still poor, and this is very dangerous as they find it difficult to accommodate those changes because they do not know their meaning (Eslamimehr *et al*., 2016).

Current studies revealed that a major source of participants' information about puberty came from their friends and peers; which were unreliable. These findings are consistent with other studies carried out in Iran (Madanipour *et al*., 2009; Ramezanzadeh *et al*., 2010).

More than half of the participants mentioned that they wanted to receive knowledge on RH from others; this means that they want to discuss together issues associated with reproductive health and learn from each other. Blazar and Kraft (Blazar & Kraft, 2017) indicated that the behaviors are developed and shared in specific groups such as peers, choir, schools, adult friends and drama clubs. These are very important findings, which suggest that reproductive health education to the population of this age group would be more successfully delivered through FDG (Borawski *et al*., 2015).

The study findings showed the need to enhance interaction and positive attitudes among teachers and adolescent girls. Almost half of the participants mentioned teachers as their source of information indicating that school is the place where adolescents spend most of their time under the care of their teachers. Teachers can play a great role in adolescents' behavior change because adolescents trust and learn from them through discussions, imitation and following role modeling. Teachers should be non-judgmental and friendly when they teach adolescents about RH (Borawski *et al*., 2015). This attitude enables them to better understand the problems of adolescents and share their RH concerns with them (Kaushal *et al*., 2015). A number of studies have shown that teachers could enhance communication with adolescents by showing empathy when interacting with them. This question-item aimed to know the sources from which adolescents get information on reproductive health (Blazar & Kraft, 2017; Borawski *et al*., 2015; Kaushal *et al*., 2015). Therefore, if teachers play their role effectively in educating adolescents on RH, most of them will be knowledgeable and have good RH.

Since PAR is systematic and rigorous, this method will enable stakeholders and researchers to explore and discover effective solutions during the research process. Using PAR will enable increased engagement and collaboration with the research participants and stakeholders. Nevertheless, the current study had several limitations. It was a qualitative study; thus, we were limited in generalizing our results. In addition, different types of research questions are required to address different types of research designs. Participants were only unmarried adolescent girls in high schools; if it had included married adolescents and out-of-school adolescent girls, it would have had outcomes that are more comprehensive.

## CONCLUSIONS

In-Depth multiple studies were conducted using adolescents' needs assessment as well as discussions with investigators about factors associated with RH promotion. This study improved our understanding of identifying factors associated with effective development, implementation, and evaluation of RH based on PAR.

Our results show that school-based RHP is an essential program for improving adolescents' knowledge and it is cost-effective in the prevention of health issues. This study showed changes among the participants through a partnership program. The findings indicate that PAR enhances RH knowledge and it appears to be a valuable approach in RH promotion. Despite limitations, our review indicates that RHP based on PAR may be an effective strategy to promote adolescents' knowledge on RH.
